# Decoding oral cancer: insights from miRNA expression profiles and their regulatory targets

**DOI:** 10.3389/fmolb.2024.1521839

**Published:** 2025-01-28

**Authors:** Xin Wang, Shuang Zhang, Shuyi Wang, Tao Cao, Hong Fan

**Affiliations:** ^1^ Department of Endodontics, The First Affiliated Hospital of Harbin Medical University, Harbin, China; ^2^ Department of Endodontics, School of Stomatology, Harbin Medical University, Harbin, China; ^3^ Stomatological Hospital of Chongqing Medical University, Chongqing, China

**Keywords:** oral cancer, miRNAs, gene expression, cell cycle, PPIs

## Abstract

Oral cancer (OC) is a prevalent malignancy with high mortality rates, largely attributed to late diagnosis and limited therapeutic advancements. MicroRNAs (miRNAs), as critical regulators of gene expression, have emerged as key players in modulating plethora of cellular mechanisms. This study analyzed miRNA and gene expression profiles in OC using publicly available datasets from the Gene Expression Omnibus (GEO) to explore their roles in tumorigenesis. A total of 23 differentially expressed miRNAs (DEmiRs) and 1,233 differentially expressed genes (DEGs) were identified. Functional annotation and pathway enrichment analyses highlighted significant involvement of DEmiRs and their target genes in cell cycle-related processes, including enrichment in the nucleus, transcription factor activity, regulation of nucleosides, nucleotide and nucleic acids, cell growth and/or maintenance, mitotic cell cycle, mitotic M-M/G1 phases an DNA replication. Furthermore, different signaling cascades such as IGF signaling, PDGF signaling and LKB1 signaling and PLK1 signaling pathways were also found associated with DEmiR-related regulation of OC progression. Protein-protein interaction (PPI) network analysis identified key molecular hubs associated with DEmiR and DEGs in OC. Notably, most of these hub genes such as NEK2, NDC80, NUF2, PLK1, SMAD2, TP53, TPX2, TTK, UBE2C, WDHD1, WTAP, YWHAZ are directly or indirectly associated with cell cycle progression, underscoring the role of DEmiRs in driving tumor proliferation and survival in OC via dysregulating cell cycle. This study offers insights into the molecular mechanisms underlying OC and highlights miRNAs as potential biomarkers and therapeutic targets to disrupt the cancerous cell cycle and improve treatment outcomes.

## 1 Introduction

Oral cancer (OC) is a malignant disease that poses significant threat to human health, ranking as the sixth most common systemic tumor worldwide (Wong and Wiesenfeld, 2018). There are approximately 275,000 new OC patients every year, accounting for around 25% of all tumors. Unfortunately, about 130,000 of these patients succumbed to death every year (Abati et al., 2020), with 67% of OC cases occurring in India, Pakistan, and other parts of South Asia (Khurshid et al., 2018). In recent years, China has also seen gradual increase in the incidence of OC. Oral squamous cell carcinoma (OSCC) is the most prevalent type of OC, accounting for approximately 95% of oral malignancies. OC is often undetectable in its early stages as early diagnosis rate is less than 10%. With the continuous advancements in surgery, radiotherapy and chemotherapy, the 5-year survival rate of OC patients has substantially improved, however, it still hovers at around 63% (Chattopadhyay et al., 2019). The occurrence of OC has been found related to smoking, drinking, and chewing areca nut (Dhanuthai et al., 2018). Additionally, infections caused by human papillomavirus (HPV) and other viruses have also been found associated with the incidence of OC (Li et al., 2020). Epidemiological studies have shown that OC is a disease caused by the combined action of genetic and environmental factors (Rodriguez-Molinero et al., 2021), whereas a recent study has pointed out that abnormal gene expression is the main factor leading to the occurrence of OC (Ramos-Garcia et al., 2021). Therefore, exploring the molecular mechanism regulating disease onset and progression in OC holds significant potential for identifying novel therapeutic targets and guiding the development of more effective treatment strategies

miRNAs are non-protein encoding single-stranded RNAs transcribed from genome and consists of 20–25 nucleotides. Widely present in eukaryotes, miRNAs exhibit characteristic hairpin structure. By complementary pairing with target mRNAs, miRNAs regulate the gene expression at the post-transcriptional level, resulting in changes in mRNA stability and protein expression (O'Brien et al., 2018). miRNAs are involved in various biological processes includingl phylogeny, cell proliferation, apoptosis, metabolism, and immune defense (Andersen and Tost, 2020). As crucial regulators of gene expression, miRNAs control the expression of almost 30% of human genes (Budakoti et al., 2021). However, numerous studies have highlighted dysregulated miRNA expression in tumor tissues a (Lai et al., 2022), consistent with the findings that 50% of miRNAs in the genome are located at fragile sites, where mutations are strongly associated with cancer development (Dejene et al., 2019). In line with these, dysregulated expression of different miRNAs have also been associated with OC over the years. For instance, elevated plasma levels of miR-146a have demonstrated diagnostic value in OSCC, where it promotes tumorigenesisby targeting multiple genes (Ghuwalewala et al., 2021). Conversely, low expression of miR-375 is significantly associated with advanced lesions, tumor size, and invasive patterns in OSCC (Zhang et al., 2017), suggesting its critical tumor inhibitory role OC. Alternatively, miRNA-196b expression is significantly higher in OSCC compared to adjacent normal tissues, and promotes migration and invasion *in vitro*, while silencing its expression can reverse the effects. These results highlighted miR-196b as a potential prognostic marker and therapeutic target for OSCC (Dioguardi et al., 2022). Higher expression of miR-382-5p in tumor-associated fibroblasts of OSCC patients has also been associated with increased migration and invasion of OSCC cells (Sun et al., 2019). Overall, existing literature suggests that miRNA plays an important role in tumorigenesis in OC. Despite this, comprehensive and systematic studies on miRNA expression profiles in OC remain scarce. To address this gap, this study analyzed miRNA expression profiles from OC and normal tissue samples available in the GEO database. Differentially expressed miRNAs (DE-miRNAs) were identified through bioinformatics methods, and their functions were explored using gene enrichment analysis. Findings of this study provide a theoretical foundation for the development of targeted therapies for OC.

## 2 Materials and methods

### 2.1 Patient’s data

OC-associated gene chip datasets were downloaded from Gene Expression Omnibus (GEO) (https://www.ncbi.nlm.nih.gov/gds), including GSE31056 (Reis et al., 2011), GSE113956 (Shi et al., 2019), and GSE124566 (Zhuang et al., 2020). GSE31056 contained gene expression profiling data of 23 OC tissues and 23 normal tissues, GSE113956 included miRNA expression profiling of serum samples from 25 OSCC patients and 15 healthy controls, and GSE124566 covered non-coding RNA profiling of 10 OC tissues and 10 adjacent normal tissues.

### 2.2 Screening of differentially expressed miRNAs (DEmiRs) and genes (DEGs)

GEO2R is a web-based tool provided by the GEO database that identifies DEGs across two or more sample groups and ranks genes based on their significance. DEmiRs in samples from GSE113956 and GSE124566, and DEGs in samples from GSE31056 were identified using GEO2R. miRNAs and Genes with a *p*-value < 0.05 and |log2 fold change (FC)| ≥ 1.5 were considered significantly differentially expressed. Specifically, genes and miRNAs with log2FC ≥ 1.5 were classified as upregulated, whereas genes and miRNAs with log2FC ≤ −1.5 were classified as downregulated. Additionally, the Venn diagram (http://bioinformatics.psb.uget.be/webtools/Venn) was used to visualize overlapping DEmiRs between the GSE113956 and GSE124566 datasets.

### 2.3 Functional annotation and pathway enrichment analysis of DEmiRs and DEGs

Target genes of DE-miRs identified from GSE113956 and GSE124566 were predicted by mir-Walk3.0 (http://mirwalk.umm.uni-hei-delberg.de/). The miR-target genes and identified DEGs were subjected to gene ontology (GO) and Kyoto Encyclopedia of Genes and Genomes (KEGG) pathway enrichment analyses. GO analysis was performed using GOseq (Release 2.12) and focused on three categories: biological processes (BP), cellular components (CC), and molecular functions (MF). KEGG pathway analysis was conducted using KOBAS 3.0. For both GO and KEGG analyses, results with a *p*-value < 0.05 were considered statistically significant.

### 2.4 Analysis of protein-protein interactions (PPIs)

PPIs were predicted using STRING (https://string-db.org/) database which evaluates the likelihood of protein interactions. The list of proteins encoded by miR-target genes and identified DEGs were uploaded to the STRING library with the interaction confidence score threshold set to a binding score >0.4. PPI networks were then constructed using Cytoscape (version 3.6.1), and the top 10 key genes were identified based on their scores calculated using the Degree algorithm. These scores were visually represented with distinct colors to highlight the relative importance of each gene in the hub network.

## 3 Results

### 3.1 Identification of DEmiRs and DEGs in OC

**FIGURE 1:**
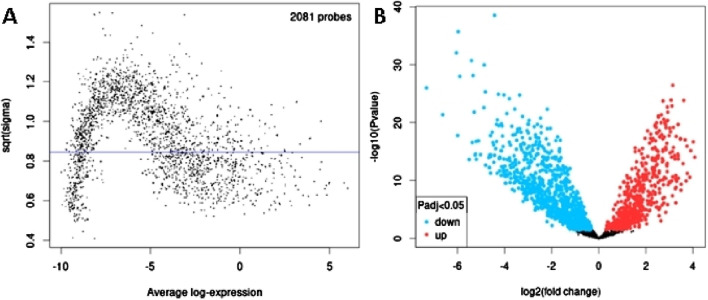
Differential expression analysis of miRNAs from GSE113956. **(A)** The relationship between the square root of variance [sqrt (σ)] and average log expression for 2081 probes. Each dot represents a probe, with variance plotted as sqrt (σ) and its corresponding average log expression. **(B)** Volcano plot showing log2 (fold change) vs. −log10 (*p*-value). Red and blue dots represent significantly upregulated and downregulated miRNAs (*p*-adjusted <0.05), respectively, while black dots are non-significant miRNAs.

**FIGURE 2:**
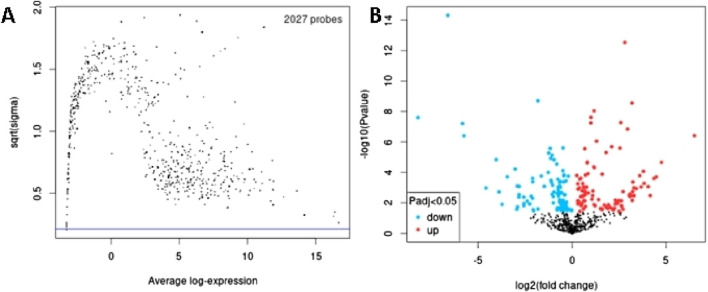
Differential expression analysis of miRNAs from GSE124566. **(A)** The relationship between the square root of variance [sqrt (σ)] and average log expression for 2027 probes. Each dot represents a probe, with variance plotted as sqrt (σ) and its corresponding average log expression. **(B)** Volcano plot showing log2 (fold change) vs. −log10 (*p*-value). Red and blue dots represent significantly upregulated and downregulated miRNAs (p-adjusted <0.05), respectively, while black dots are non-significant miRNAs.

**FIGURE 3:**
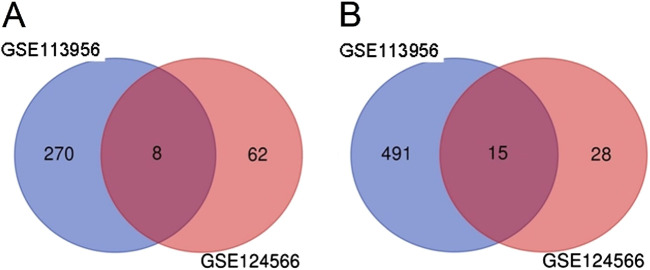
Common DEmiRs in GSE113956 and GSE124566. **(A)** Venn diagram showing number of overlapping upregulated miRNAs in GSE113956 and GSE124566. **(B)** Venn diagram showing number of overlapping downregulated miRNAs in GSE113956 and GSE124566.

In order to identify DEmiRs associated with OC, we used GEO2R to screen DE-miRNAs between OC patients and respective controls in GSE113956 and GSE124566. The screening results are shown in Figures 1, 2 respectively. There were 2,081 probes (miRNAs) detected in samples from GSE113956 (Figure 1A), out of which 787 were differentially expressed between OC and control group, including 278 upregulated (red dots) and 506 downregulated (blue dots) DEmiRs (Figure 1B). On the other hand, there were 2,027 probes (miRNAs) detected in samples from GSE124566 (Figure 2A), out of which 113 were differentially expressed between OC and control group, including 70 upregulated (red dots) and 43 downregulated (blue dots) DEmiRs (Figure 2B). Out of 278 upregulated DEmiRs in GSE113956 and 70 upregulated DEmiRs in GSE124566, 8 DEmiRs, namely, hsa-miR-4778-5p, hsa-miR-299-3p, hsa-miR-3138, hsa-miR-4419a, hsa-miR-142-5p, hsa-miR-454-3p, hsa-miR-625-5p, and hsa-miR-142-3p, were commonly upregulated (Figure 3A; Table 1). On the other hand, Out of 506 downregulated DEmiRs in GSE113956 and 43 downregulated DEmiRs in GSE124566, 15 DEmiRs, namely, hsa-miR-513b, hsa-miR-744-5p, hsa-miR-205-5p, hsa-miR-375, hsa-miR-1281, hsa-miR-378a-5p, hsa-miR-29c-5p, hsa-miR-429, hsa-miR-4647, hsa-miR-3188, hsa-miR-204-5p, hsa-miR-338-3p, hsa-miR-200a-3p, hsa-miR-1183, and hsa-miR-513c-5p, were commonly downregulated (Figure 3B; Table 2). Overall, these results show that numerous miRNAs are differentially expressed in OC compared to normal control tissues, suggesting their key role in tumorigenesis and progression in OC.

**TABLE 1:** 8 commonly upregulated DEmiRs in GSE113956 and GSE124566.

Name of miRNA	*p*-Value	t value	B Value	logFC
hsa-miR-4778-5p	2.59 × 10^−14^	11.34307	22.3652	2.410978
hsa-miR-299-3p	1.74 × 10^−6^	5.56121	4.3472	2.222,956
hsa-miR-3138	7.00 × 10^−4^	3.662	−1.5122	1.816605
hsa-miR-4419a	8.16 × 10^−11^	9.18541	16.0199	3.270167
hsa-miR-142-5p	1.29 × 10^−4^	4.22039	0.12	2.564011
hsa-miR-454-3p	2.26 × 10^−4^	4.03903	−0.4215	1.954795
hsa-miR-625-5p	6.98 × 10^−20^	16.51311	35.1841	3.165281
hsa-miR-142-3p	9.31 × 10^−4^	3.56454	−1.7855	2.640014

**TABLE 2:** 15 commonly downregulated DEmiRsin GSE113956 and GSE124566.

Name of miRNA	*p*-Value	t value	B Value	logFC
hsa-miR-513b	4.20 × 10^−3^	−3.25662	−2.46861	−2.57
hsa-miR-744-5p	1.55 × 10^−2^	−2.66143	−3.71001	−2.57
hsa-miR-205-5p	2.05 × 10^−1^	−1.31416	−5.97266	−1.949
hsa-miR-375	2.50 × 10^−8^	−9.12791	9.417,116	−8.194
hsa-miR-1281	1.29 × 10^−1^	−1.70471	−5.42688	−1.51
hsa-miR-378a-5p	2.66 × 10^−2^	−2.40631	−4.20935	−2.94
hsa-miR-29c-5p	1.18 × 10^−2^	−2.78723	−3.45547	−2.256
hsa-miR-429	9.49 × 10^−2^	−1.75914	−5.34242	−2.05
hsa-miR-4647	6.20 × 10^−8^	−8.6025	8.524,957	−5.826
hsa-miR-3188	3.72 × 10^−2^	−3.31137	−2.3505	−2.17
hsa-miR-204-5p	3.95 × 10^−7^	−7.58856	6.695,311	−5.746
hsa-miR-338-3p	3.76 × 10^−2^	−3.25953	−2.46233	−2.825
hsa-miR-200a-3p	1.36 × 10^−1^	−1.55715	−5.64598	−1.527
hsa-miR-1183	1.26 × 10^−1^	−1.72066	−5.40233	−2.195
hsa-miR-513c-5p	1.44 × 10^−5^	−5.80195	3.1206	−4.038

**FIGURE 4:**
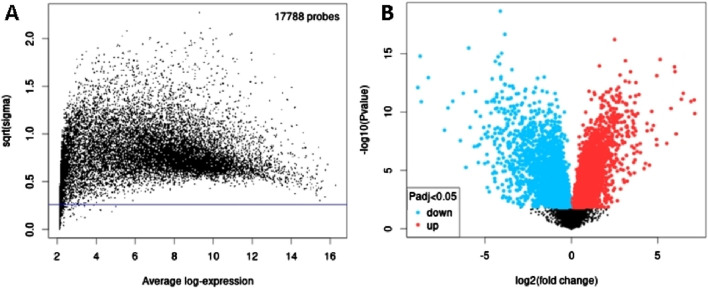
Differential expression analysis of mRNAs from GSE31056. **(A)** The relationship between the square root of variance [sqrt (σ)] and average log expression for 17,788 probes. Each dot represents a probe, with variance plotted as sqrt (σ) and its corresponding average log expression. **(B)** Volcano plot showing log2 (fold change) vs. −log10 (*p*-value). Red and blue dots represent significantly upregulated and downregulated mRNAs (p-adjusted <0.05), respectively, while black dots are non-significant mRNAs.

Next, to identify DEGs associated with OC, we used GEO2R to screen DEGs between OC patients and controls in GSE31056. There were 17,788 probes detected in samples from GSE31056 (Figure 4A),out of which 1,233 were differentially expressed betweenOC and control group, including 505 upregulated (red dots) and 728 downregulated (blue dots) DEGs (Figure 4B).

### 3.2 Functional annotation and pathway enrichment analysis of downregulated DEmiRs and upregulated DEGs

**FIGURE 5:**
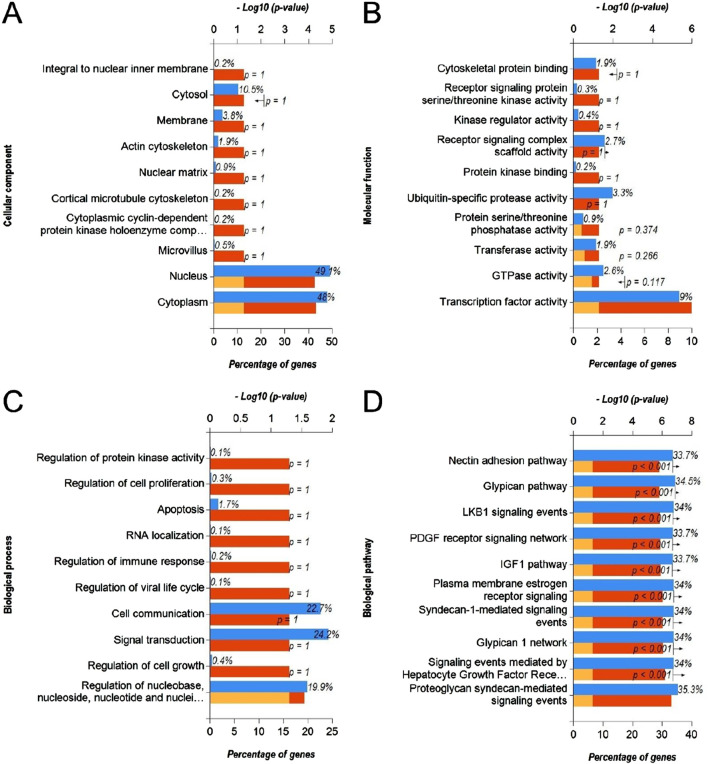
Functional annotation and pathway enrichment analysis of gene targets of downregulated DEmiRs in OC. **(A–D)** Bar-graphs showing enriched cellular compartments **(A)**, molecular functions **(B)**. Biological processes **(C)** and KEGG biological pathways **(D)** associated with gene targets of downregulated DEmiRs in OC. Blue bar shows percentage of genes enriched from each annotation, red bar shows −log10 (*p*-value), and yellow bar shows –log10 (FDR).

**FIGURE 6:**
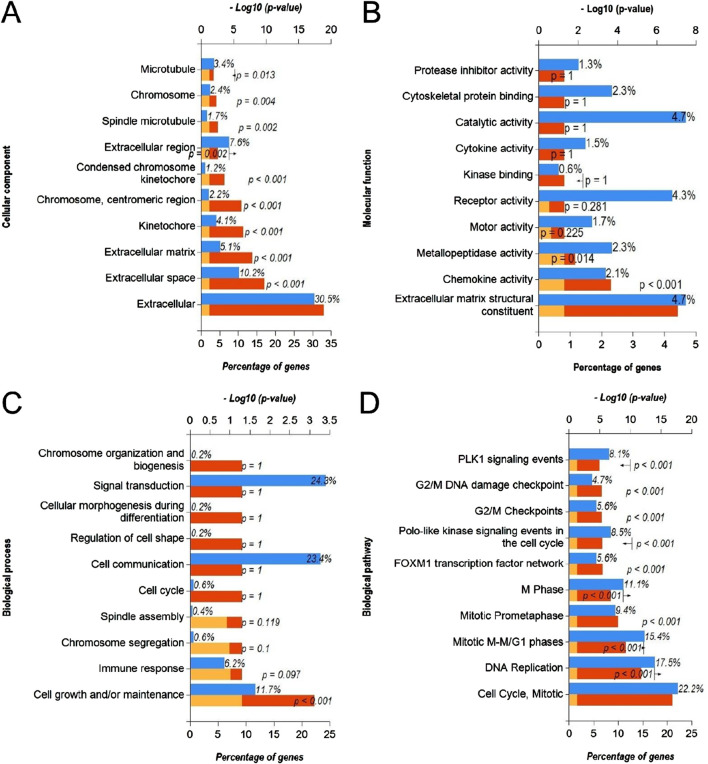
Functional annotation and pathway enrichment analysis of upregulated DEGs in OC. **(A–D)** Bar-graphs showing enriched cellular compartments **(A)**, molecular functions **(B)**. Biological processes **(C)** and KEGG biological pathways **(D)** associated with gene targets of upregulated DEGs in OC. Blue bar shows percentage of genes enriched from each annotation, red bar shows −log10 (*p*-value), and yellow bar shows –log10 (FDR).

In order to identify the molecular mechanisms regulated by DEmiRs in OC, we first predicted the target genes of downregulated DEmiRs by miR-Walk 3.0 tool. GO analysis (CC, MF and BP) of target genes of downregulated DEmiRs in OC revealed that these genes were significantly enriched in cytoplasm and nucleus (Figure 5A), and were significantly associated with transcription factor activity (Figure 5B) and regulation of nucleobase, nucleoside, nucleotide, and nucleic acid metabolic processes (Figure 5C). In addition, KEGG pathway enrichment analysis revealed that these genes were significantly associated with following pathways: proteoglycan syndecan-mediated, those mediated by HGF-1, Glypican 1 network, syndecan-1-mediated, plasma membrane estrogen receptor, IGF1 pathway, PDGF receptor, LKB1, glypican pathway, and nectin adhesion (Figure 5D). Notably, miRNA expression inversely correlates with the expression their target genes. Therefore, to validate above findings, we performed GO and KEGG enrichment analyses with upregulated DEGs in OC to determine whether they are enriched in similar pathways. GO analysis of upregulated DEGs in OC revealed that these genes were significantly enriched in different CCs including extracellular, extracellular space, extracellular matrix, kinetochore, chromosome, centromeric region, condensed chromosome kinetochore, extracellular region, spindle microtubule, chromosome, and microtubule (Figure 6A). In addition, downregualted DEGs in OC were significantly associated with extracellular matrix structural constituent, chemokine activity, and metallopeptidase activity (Figure 6B), and cell growth and/or maintenance processes (Figure 6C). Furthermore, KEGG pathway enrichment analysis revealed that these genes were significantly associated with following pathways: cell cycle, mitotic, DNA replication, mitotic M-M/G1 phases, mitotic prometaphase, M phase, FOXM1 transcription factor network, polo-like kinase (PLK), G2/M Checkpoints, G2/M DNA damage checkpoint, and PLK1 signaling events (Figure 6D). Overall, as different cell cycle related terms were primarily enriched in both analyses, these results revealed that downregulated DEmiRs and upregulated DEGs converge on hyperactive cell cycle progression in OC via their involvement in different signaling cascades.

### 3.3 Functional annotation and pathway enrichment analysis of upregulated DEmiRs and downregulated DEGs

**FIGURE 7:**
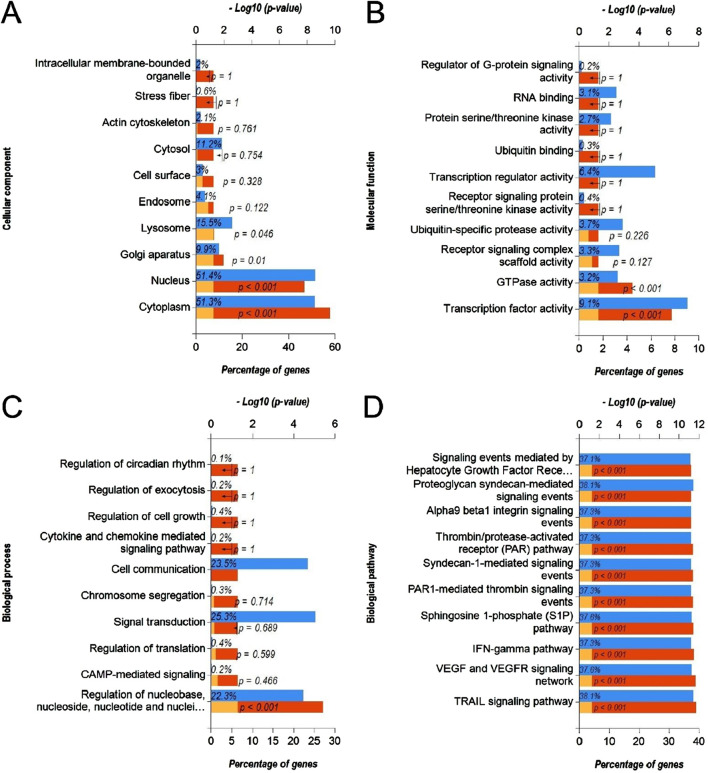
Functional annotation and pathway enrichment analysis of gene targets of upregulated DEmiRs in OC. **(A–D)** Bar-graphs showing enriched cellular compartments **(A)**, molecular functions **(B)**. Biological processes **(C)** and KEGG biological pathways **(D)** associated with gene targets of upregulated DEmiRs in OC. Blue bar shows percentage of genes enriched from each annotation, red bar shows −log10 (*p*-value), and yellow bar shows –log10 (FDR).

**FIGURE 8:**
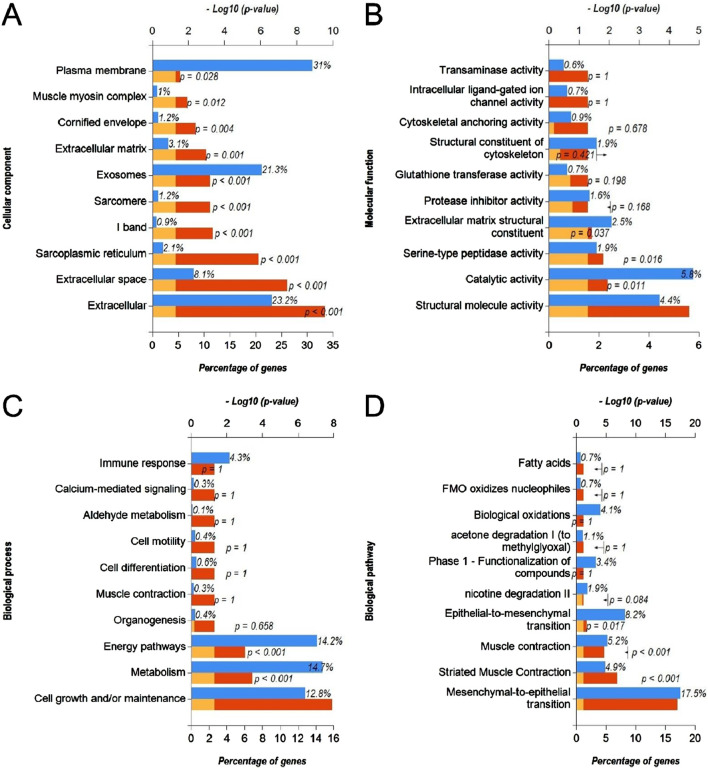
Functional annotation and pathway enrichment analysis of downregulated DEGs in OC. **(A–D)** Bar-graphs showing enriched cellular compartments **(A)**, molecular functions **(B)**. Biological processes **(C)** and KEGG biological pathways **(D)** associated with gene targets of downregulated DEGs in OC. Blue bar shows percentage of genes enriched from each annotation, red bar shows −log10 (*p*-value), and yellow bar shows –log10 (FDR).

Next, we predicted the target genes of upregulated DEmiRs by miR-Walk 3.0 tool. GO analysis (CC, MF and BP) of target genes of upregulated DEmiRs in OC revealed that these genes were significantly enriched in cytoplasm, nucleus, golgi apparatus and lysososmes (Figure 7A), and were significantly associated with transcription factor activity, GTPase activity (Figure 7B), and regulation of nucleobase, nucleoside, nucleotide, and nucleic acid metabolic processes (Figure 7C). In addition, KEGG pathway enrichment analysis revealed that these genes were significantly associated with following pathways: TRAIL, VEGF and VEGFR, IFN-gamma, sphingosine 1-phosphate, PAR1-mediated thrombin, syndecan-1-mediated, thrombin/PAR, Alpha-9 beta-1 integrin, proteoglycan syndecan-mediated, and those mediated by Hepatocyte Growth Factor l (HGF-1) (Figure 7D). Next, we performed GO and KEGG enrichment analyses with upregulated DEGs in OC to determine whether they are enriched in similar pathways. GO analysis of downregulated DEGs in OC revealed that these genes were significantly enriched in different CCs including extracellular, extracellular space, sarcoplasmic reticulum, l band, sarcomere, exosomes, extracellular matrix, cornified envelope, muscle myosin complex, plasma membrane (Figure 8A). In addition, downregualted DEGs in OC were significantly associated with structural molecule activity, catalytic activity, serine-type peptidase activity, extracellular matrix structural constituen, protease inhibitor activity, glutathione transferase activity, structural constituent of cytoskeleton, cytoskeletal anchoring activity, intracellular ligand-gated ion channel activity, and transaminase activity (Figure 8B), and cell growth and/or maintenance, metabolism, energy pathways, organogenesis, muscle contraction, cell differentiation, cell motility, aldehyde metabolism, calcium-mediated signaling and immune response (Figure 8C). Furthermore, KEGG pathway enrichment analysis revealed that these genes were significantly associated with following pathways: mesenchymal-to-epithelial transition, striated muscle contraction, muscle contraction (Figure 8D). Overall, the results from these analysis also hinted that upregulated DEmiRs and downregulated DEGs are associated with cell cycle regulation to some extent. However, downregulated DEGs may be primarily regulated by some alternate mechanism than by upregulated DEmiRs in OC as molecular mechanisms and pathways associated with upregulated DEmiRs were distinct from those associated with downregulated DEGs, such as downregulation of TRAIL signaling and IFN-gamma pathway (Figure 7D) highlight immune evasion properties of OC, whereas downregulation of mesenchymal-to-epithelial transition (Figure 8D) hints to progression towards aggressive disease state, all contributing towards cancer progression in OC.

### 3.4 PPI network analysis of DEmiR-regulated genes and DEGs

**FIGURE 9:**
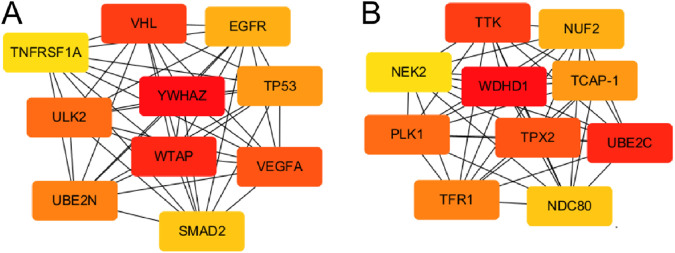
PPI networks of miRNA-regulated and DEG-enriched hubs in OC. **(A)** PPI network of hub genes regulated by DEmiRs in OC. **(B)** PPI network of hub genes among DEGs in OC. Color code represent the importance of each gene based on the Degree sorted scores from Cyctoscape with red being the most important hubs.

In order to identify the molecular hubs associated with miRNA-driven molecular mechanisms in OC, we developed PPI network of DEmiR regulated genes. This network had 1,768 nodes and 14,290 edges, and an average local clustering coefficient (LCC) of 0.238. The top 10 key genes were tyrosine 3/tryptophan 5-monooxygenase-activating protein (YWHAZ), WTAP, VHL syndrome, vascular endothelial growth factor A (VEGFA), uncoordinated 51-like kinase 2 (ULK2), ubiquitin coupled enzyme 2N (UBE2N), tumor protein p53 (TP53), epidermal growth factor receptor (EGFR), SMAD homolog 2 (SMAD2), and tumor necrosis factor receptor superfamily member 1A (TNFRSF1A) (Figure 9A). On the other hand, we also constructed PPI network of DEGs in OC. There were 1,175 nodes and 10,780 edges, and an average LCC of 0.379. The top 10 key genes were WD repetition and HMG frame DNA-binding Protein 1 (WDHD1), ubiquitin coupling enzyme 2C (UBE2C), tyrosine threonine kinase (TTK), targeting protein for *Xenopus* kinesin-like protein2 (TPX2), PLK1, transferrin receptor 1 (TFR1), teneurin carboxy-terminal associated peptide (TCAP-1), NUF2, nuclear division cycle 80 (NDC80), and centrosome associated kinase 2 (NEK2) (Figure 9B). Notably, most of these identified hubs are directly associated with cell cycle progression suggesting a close link between miRNA dysregulation and cell cycle progression in OC.

## 4 Discussion

Currently, the treatment outcomes for OC are unsatisfactory, leading to poor prognosis and lower survival rates for patients with OC (Komlos et al., 2021). Therefore, exploring the molecular mechanism of OC has positive guiding significance for the development of OC targeted therapy. miRNAs, as non-coding RNAs, play a positive role in gene regulation and are currently recognized as cancer intervention targets and prognostic and diagnostic tools (Yao et al., 2022). Studies have identified different tumor suppressor and oncogenic miRNAs in OC (El Aamri et al., 2020). For instance, miR-24 and miR-146a were significantly upregulated in serum of OC patients, among which miR-146a enhanced carcinogenicity through targeted regulation of NUMB and other genes (Arshinchi Bonab et al., 2022). Here, we analyzed OC-related miRNA and gene expression patient data from GEO database to identify the miRNA regulators of diseases. The differential miRNA analysis showed that there were 23 common DE-miRNAs (8 upregulated and 15 downregulated) in the GSE113956 and GSE124566, and the logFC of hsa-miR-375 was the minimum −8.194, suggesting that hsa-miR-375 was the most downregulated. Notably, Tu et al. found that miR-375 expression was greatly reduced in OSCC patients, and this decrease was related to OSCC lesions, tumor size and invasion pattern (Tu et al., 2022). Similarly, Zhao et al. has reported that miR-375 expression is low in liver cancer, which can serve as a biomarker for tumor onset and progression (Zhao et al., 2020).

Through the GO function analysis of miRNA, it was found that DEmiRs target genes were enriched in different cellular compartment includingcytoplasm, nucleus, golgi apparatus and lysosomes (Figures 5A, 7A). miRNAs biogenesis takes place in nucleus and cytoplasm, and they primarily function in cytoplasm. In addition, it has been suggested that DE-miRs are related to Golgi apparatus where they are responsible for protein processing and their transport to specific sites of cells or extracellular space (Liu et al., 2022). On the other hand, molecular functions and biological processes as well as KEGG pathways associated with DEmiRs were mainly enriched in transcription factor activity, cell growth and/or maintenance, and nucelobase, nucleoside, nucleotide, and nucleic acid metabolism (Figures 5, 7). Such results suggest that DE-miRNAs are mainly involved in cell cycle regulation. These results are in line with the findings that miRNAs play critical role in cell cycle progression and contribute to tumor onset, progression and metastasis (Chivukula and Mendell, 2008; Mens and Ghanbari, 2018). Functional pathway enrichment analysis of DEGs also hinted towards aberrant cell cycle progression in OC compared to normal controls (Figure 6) aligning with previous findings.

In addition, DE-miRs were also found to be involved in TRAIL, VEGF and VEGFR, alpha nine beta 1 integrin, plasma membrane estrogen receptor, and PDGF receptor signaling (Figure 7D). These mechanisms could serve as interlink between DEmiR-driven regulation of cell cycle control. TRAIL is a member of tumor necrosis factor superfamily, widely distributed in normal tissues such as lung, spleen, kidney, and peripheral lymphocytes, and can selectively induce tumor cell apoptosis (Yuan et al., 2018). TRAIL can specifically activate apoptosis signaling pathways after binding to death receptors on cell membranes (Singh et al., 2021). The results suggest that TRAIL may be correlated with OC. Cardoso Alves et al. (2021) found that TRAIL is upregulated in bile duct cancer tissues, but the high expression of TRAIL do not produce significant inhibitory effect on bile duct cancer cells (Razeghian et al., 2021). VEGF and its receptor VEGFR, as transcription regulatory factors, aid in alleviating hypoxia and nutritional status in tumor cells by stimulating the formation of blood vessels. Apte et al. (2019). At the same time, VEGF/VEGFR signaling is associated withhigh proliferation, invasion, metastasis and migration in cancer cells (Melincovici et al., 2018). Besides, it has also been reported to be involved in drug resistance mechanisms (Itatani et al., 2018). After binding to its receptor, estrogen activates intracellular signals and thus participates in the regulation of protein function (Chen et al., 2018). Current studies have found that plasma membrane estrogen receptor is associated with gynecological tumors such as breast cancer, endometrial cancer, and cervical cancer (Wan et al., 2021). Meanwhile, some researchers have pointed out that plasma membrane estrogen receptor is associated with the occurrence of lung cancer, esophageal cancer, and other cancers (Siveen et al., 2017). Platelet-derived growth factor (PDGF) regulates the differentiation and proliferation of fibroblasts, and is involved in cell fibrosis through autocrine and paracrine signaling, promoting the proliferation of fibroblasts, resulting in abnormal performance of interstitial collagen, and eventually leading to tissue fibrosis (Guerit et al., 2021). PDGF has been found significantly upregulated in OC cells where it activates NF-kB by regulating the level of miR-26a-5p, promoting the activation of fibroblasts, and thus contributing to occurrence of OC (Zou et al., 2022).

In this study, the top 10 key DEmiR target hubs in thePPI network were YWHAZ, WTAP, VHL, VEGFA, ULK2, UBE2N, TP53, EGFR, SMAD2, and TNFRSF1A (Figure 9), among which WTAP, VHL, TP53 and SMAD2 were significantly downregulated in OC tissues compared to controls whereas the rest were upregulated. YWHAZ protein is an important inhibitor of apoptosis, mainly distributed in the cytoplasm, involved in cell proliferation, adhesion, signal transduction and apoptosis. It is highly expressed in gastric, ung, breast, and cervical cancers andgliomas, where it is involved in the occurrence, progression, and metastasis of tumors (Gan et al., 2020; Zhao et al., 2021). WTAP, a widely expressed nuclear protein, and is linked toh regulating the apoptosis and cell cycle. It can work as a splice body to participate in the selective splicing of precursor mRNA and play a critical role in body growth and development (Chen et al., 2019). WTAP is associated with the occurrence of hepatocellular carcinoma, lung adenocarcinoma, colon, ovarian, and bile duct cancers (Li et al., 2022). WTAP can inhibit the growth and proliferation of OC cells by inhibiting their glucose metabolism, and then affect the process of OC (Wei et al., 2021). VHL can play a tumor suppressor role through its encoded pVHL and regulate cell cycle, cell differentiation, placental angiogenesis and embryonic development (Yang et al., 2022). Its expression is significantly reduced in angioblastoma, renal cell carcinoma, pancreatic cancer, and other tumors (Stransky et al., 2022). Low expression of VHL has been found associated with tumor growth and nvasion in OSCC (Jonasch et al., 2021). VEGFA is a member of VEGF family, which is critical in tumor angiogenesis, the division and proliferation of vascular endothelial cells and lymphatic endothelial cells in tumor tissues (Kieran et al., 2012). ULK2 is involved in the autophagy process by promoting the phosphorylation level of downstream proteins, and ULK2 expression is significantly upregulatedin breast cancer and, hepatocellular and esophageal carcinomas (Tsang et al., 2020).

This work revealed that the top 10 key DEG hubs in OC were WDHD1, UBE2C, TTK, TPX2, PLK1, TFR1, TCAP-1, NUF2, NDC80, and NEK2 (Figure 9), all of which were significantly upregulated in OC tissue compared to control. WDHD1 is a protein containing the WD 40 domain and a DNA-binding protein 1 with a high mobility group. WDHD1 is a DNA replication initiation factor that is involvedin regulating protein signal transduction, transcription, cell apoptosis and other processes. WDHD1 is highly expressed in patients who suffer from pancreatic, liver, breast, lung and esophageal cancers, and can be used as a tumor marker (Wu et al., 2022). WDHD1 can activate PI3K/Akt signal transduction and promote epithelial-to-mesenchymal transition which leads to cell invasion and metastasis, and provides basis of malignant transformation. WDHD1 is highly expressed in tubular SCC tissues and is related to occurrence, progression, invasion, and metastasis of esophageal SCC (Liu et al., 2019) In addition, WDHD1 has been shown to induce the occurrence and further development of ESCC by activating PI3K/Akt signal transduction (Ertay et al., 2020). As a cancer suppressor gene, UBE2C importantly regulates the cell cycle. Its expression is significantly increased in brain, breast, colorectal, esophagal, gastric, and nasopharyngeal where its expression is positively correlated with tumor grade and poor prognosis (Dastsooz et al., 2019). TTK is a component of the spindle assembly checkpoint, which phosphorylates serine, threonine, and tyrosine residues, and promotes metaphase mitotic chromosome alignment. TTK expression is increased in prostate, thyroid, esophagal, and other cancer tissues and is correlated with postoperative recurrence and poor prognosis (Qi et al., 2021). TPX2 is a microtubule-related protein that is strictly regulated by cell cycle and plays an important in cell division by promoting cell proliferation and affecting cell apoptosis. Its expression is significantly upregulated in lung SCC and salivary gland carcinomas. It has been suggested that TPX2 enhances tumor cell invasion by up-regulating cadherin levels (Neumayer et al., 2014). PLK1 is closely related to cell division and DNA damage repair (Shah et al., 2019). It is often upregulated in lung, prostate and bladder cancer, and malignant melanomas, where it is associated with poor prognosis. PLK1 may interact with P53 to inhibit P53 checkpoint and promote apoptosis (Iliaki et al., 2021). TFR1 is a type II transmembrane protein involved in iron absorption, ion transport, and immune regulation. It has been found associated with the occurrence of cervical cancer and liver cancer where it regulate cancer stemness (Xiao et al., 2020; Huang et al., 2022).

Cell cycle progression is key to maintaining normal cellular growth, division, and homeostasis. Dysregulation of this tightly controlled process often leads to uncontrolled proliferation, a hallmark of cancer (Hanahan, 2022). Notably, most of the genes identified as hub genes in the PPI networks (Figure 9) are closely associated with the regulation of cell cycle progression, highlighting their potential roles in tumorigenesis. These findings align with numerous studies demonstrating that hyperactive cell cycle progression is a driving force behind tumor progression (Suski et al., 2021). Aberrant activation of cyclins, cyclin-dependent kinases (CDKs), and their regulatory pathways not only accelerates the G1/S and G2/M transitions but also circumvents key cell cycle checkpoints designed to maintain genomic integrity. Such deregulation enables tumor cells to proliferate uncontrollably, resist apoptosis, and adapt to hostile microenvironments (Ding et al., 2020). Furthermore, the identification of cell cycle-related hub genes underscores their potential as therapeutic targets, as their inhibition may disrupt the proliferative advantage of tumor cells and restore control over cell division (Otto and Sicinski, 2017; Suski et al., 2021). Thus, the convergence of hub genes on cell cycle regulation emphasizes the critical role of cell cycle dysregulation in cancer biology, while providing insights into miRNA-based intervention strategies that target these molecular mechanisms to impede tumor progression in OC.

## 5 Conclusion

This study comprehensively analyzed miRNA and gene expression profiles in OC to elucidate their roles in tumor progression, with a particular focus on cell cycle dysregulation. The identification of 23 DEmiRs and 1,233 DEGs revealed significant enrichment of these molecules in processes critical to cell cycle regulation, including mitotic progression, DNA replication, and checkpoint control. These findings align with existing evidence that cell cycle dysregulation is a hallmark of cancer, driving uncontrolled proliferation and tumor growth. Key hub genes identified in the PPI network analysis, such as PLK1, TP53, NEK2, UBE2C, and WDHD1, were strongly associated with hyperactive cell cycle pathways. These genes play pivotal roles in the regulation of G1/S and G2/M transitions, chromosome alignment, and spindle formation, underscoring their importance in maintaining genomic stability. Dysregulated miRNAs were also implicated in modulating these processes, either by downregulating tumor suppressor genes or upregulating oncogenic pathways. Notably, pathways such as VEGF, PDGF, and TRAIL signaling were enriched, providing additional insights into how miRNAs influence the tumor microenvironment and immune evasion mechanisms. This study highlights the convergence of miRNA dysregulation and cell cycle aberrations as central drivers of OC progression. The identification of specific miRNAs and genes as potential biomarkers and therapeutic targets offers promising avenues for precision medicine. Targeting the cell cycle-related pathways and the regulatory roles of miRNAs could lead to novel treatment strategies that disrupt the proliferative advantage of cancer cells while improving patient outcomes. These findings underscore the need for further research to translate these molecular insights into clinical applications for OC management.

## Data Availability

The datasets presented in this study can be found in online repositories. The names of the repository/repositories and accession number(s) can be found in the article/supplementary material.
